# Experiences of primary care professionals providing healthcare to recently arrived migrants: a qualitative study

**DOI:** 10.1136/bmjopen-2016-012561

**Published:** 2016-09-22

**Authors:** Antje Lindenmeyer, Sabi Redwood, Laura Griffith, Zaheera Teladia, Jenny Phillimore

**Affiliations:** 1 Institute of Applied Health Research, University of Birmingham, Birmingham, UK; 2 School of Social and Community Medicine, University of Bristol, Bristol, UK; 3 Health Services Management Centre, College of Social Sciences, University of Birmingham, Birmingham, UK; 4 Institute of Research into Superdiversity, College of Social Sciences, University of Birmingham, Birmingham, UK

**Keywords:** PRIMARY CARE, QUALITATIVE RESEARCH

## Abstract

**Objectives:**

The main objectives of the study were to explore the experiences of primary care professionals providing care to recent migrants in a superdiverse city and to elicit barriers and facilitators to meeting migrants' care needs. This paper focuses on a strong emergent theme: participants' descriptions and understandings of creating a fit between patients and practices.

**Design:**

An exploratory, qualitative study based on the thematic analysis of semistructured interviews.

**Setting and participants:**

A purposive sample of 10 practices. We interviewed 6 general practitioners, 5 nurses and 6 administrative staff; those based at the same practice opted to be interviewed together. 10 interviewees were from an ethnic minority background; some discussed their own experiences of migration.

**Results:**

Creating a fit between patients and practice was complex and could be problematic. Some participants defined this in a positive way (reaching out, creating rapport) while others also focused on ways in which patients did not fit in, for example, different expectations or lack of medical records. A small but vocal minority put the responsibility to fit in on to migrant patients. Some participants believed that practice staff and patients sharing a language could contribute to achieving a fit but others outlined the disadvantages of over-reliance on language concordance. A clearly articulated, team-based strategy to create bridges between practice and patients was often seen as preferable.

**Conclusions:**

Although participants agreed that a fit between patients and practice was desirable, some aimed to adapt to the needs of recently arrived migrants, while others thought that it was the responsibility of migrants to adapt to practice needs; a few viewed migrant patients as a burden to the system. Practices wishing to improve fit might consider developing strategies such as introducing link workers and other ‘bridging’ people; however, they could also aim to foster a general stance of openness to diversity.

**Strengths and limitations of this study**

- This study is, to the best of our knowledge, the first seeking the perspectives of primary care providers on caring for a range of recent migrants (not just asylum seekers) in the UK.
- Primary care providers gave detailed reflections on barriers and facilitators of providing care to recent migrants; they discussed wider interactions between migrants and practice staff which enabled us to understand the importance of a good fit between patients and practice.
- Participants who agreed to be interviewed had a strong interest in the topic which contributed to the richness of the data but is also a limitation.
- As this is an exploratory study with a relatively small sample size, more research is needed to confirm and further contextualise the findings.

## Introduction

The advent of migration-driven superdiversity means more migrants are arriving from more places to more places in the UK, than ever before. For example, the 2011 Census found that 238 313 Birmingham residents were born outside the UK; of these, around 45% had arrived during the past decade. These individuals are diverse across a wide range of variables, including faith, immigration status, class and education, within and between ethnic groups.^1^ The challenges associated with provision of appropriate services for such diverse populations have been widely acknowledged.^2–4^ Although not all recently arrived migrants register with National Health Service (NHS) primary care services, many do; some will be encountering a primary care service for the first time in their lives.^5^ Their experiences and expectations of healthcare are likely to differ from established ethnic minority patients who are familiar with primary care and more likely to be confident in speaking English. Established migrants may also have easier access to a practice where their language is spoken or where they can consult with a doctor from a similar ethnic background.^6^ This is frequently not the case for new migrants who now arrive from almost every country in the world, often without an established community to support them. Research seeking the perspectives of migrants (especially asylum seekers) indicates that they may find it difficult to understand the system in which the general practitioner (GP) acts as gatekeeper to other services, with many feeling that their concerns had not been taken seriously by the GP.^7–10^

To explore primary care professionals' (PCPs) experiences, we are taking a ‘broad’ approach to experience caring for migrants as there is some existing work on engagement with asylum seekers and refugees in the UK;^11^ a wider focus is also likely to pick up on issues related to caring for a very diverse patient population that cut across groups. Focusing on the experience of caring for recent migrants is important as uptake of GP registration by recent entrants to the UK has been low;^12^ engagement with primary care is a process that can take some time and may depend on factors such as the availability of friends to act as navigators^13^ or budget flights enabling access to healthcare providers ‘back home’.^10^ Research with PCPs from Scandinavian countries outlines a range of challenges for primary care, including the sociocultural diversity of the migrant patient population, language barriers and assisting migrants in navigating an unfamiliar healthcare system.^14^ ^15^ A Delphi study from Canada identified language challenges, hassle, limited availability of staff and financial loss as factors that limit acceptance of migrants by PCPs,^16^ while another Canadian study found that GPs experienced a dilemma between ‘ideal’ care (eg, taking a lot of time, involving an interpreter) and the ‘real-world’ care they provided to migrants with limited English.^17^ Our study aimed to expand on these findings by eliciting the perspectives of PCPs in the superdiverse city of Birmingham. Our initial focus was on barriers and facilitators to providing high-quality healthcare to migrant patients; this paper will centre on a core emerging theme developed from the study, that of creating a fit between practice and patients.

## Methods

For this exploratory, qualitative study, we purposively recruited 10 practices in Birmingham. We started with two practices that had a strong interest in migrant health research: Practice 1 were looking for ways to cope with waves of very different migrants as they were situated in an ‘escalator area’, that is, an area which recent migrants move to and then move on, replaced by other new arrivals,^18^ while Practice 2 provided care for many asylum seekers and refugees. Further practices were recruited through a snowball strategy using contacts acquired through the University of Birmingham, migrant health networks and participants from previous research. A researcher also delivered an invitation letter in person to every primary care practice in the city which resulted in four members of admin staff participating (Practices 4, 5 and 8). Most practices were situated in areas of high diversity, with a mixture of long-established and recently arrived migrant patients. We aimed to recruit some practices in low-diversity areas for comparison and were able to also involve three practices with a mostly White British population (see table 1).

**Table 1:** Practices and participants

ID	Practice size*	Diversity†	GP	Nurse	Admin
Practice 1	Medium	High	1	1	1
Practice 2	Large	High	1	2	
Practice 3	Large	High	1	1	1
Practice 4	Medium	High			1
Practice 5	Small	Low			1
Practice 6	Small	High	1		
Practice 7	Medium	Low		1	
Practice 8	Small	High			2
Practice 9	Medium	Low	1		
Practice 10	Medium	High	1		
Total			6	5	6

*Size was derived from the publicly available practice list size where small equals <5000 patients; medium between 5000 and 10 000 patients and large >10 000 patients.

†Diversity was self-reported by interviewees and resonated with our own knowledge of the local areas the practices were situated in.

Six GPs, five practice nurses and six administrative members of staff volunteered to take part; those based at the same practice opted to be interviewed together, for example, in a lunchtime session. Although we did not purposively select respondents for ethnicity, five GPs, two nurses and three admin staff were from an ethnic minority background and some discussed their own experience of migration or healthcare systems other than the NHS. Interviews focused on challenges in caring for migrants and facilitators for providing good care. We asked participants to focus on recent migrants (defined as having been in the UK for <5 years). These patients were often discussed within the context of or in contrast to long-established migrants.

Interviews were transcribed and entered into N-vivo to enable data management and retrieval. We conducted two rounds of thematic analysis following Braun and Clarke's 6-step process.^19^ Analysis was led by AL and ZT; the other authors provided critical feedback on the developing analysis. The first phase focused on barriers and facilitators to good care. We found that while there were individual-related, provider-related or system-related factors (outlined in box 1), these were also linked with more overarching points made by participants around the relationship between patients and practice staff. Therefore, we added a second phase of analysis focusing on the nature and context of interactions between PCPs and migrant patients; from this, the concept of the fit between patients and practice emerged as a central theme. To make our results more accessible to applied health research and policy, we formulated themes as individual statements that could be linked together to show how patterns of interaction between practice and patients could lead to an increased or a decreased sense of there being a fit.^20^

Box 1 **Barriers and facilitators to successful care**

Barriers to Successful Care

- Language barriers:
  Cases where people clearly need interpreters (see below)
  Misunderstandings when some migrants and practitioners attempt to communicate in English
- Interpreting services:
  Generally available at 48 hours' notice: difficulties arise outside the ‘normal’ consultation, for example, emergencies, telephone consultations, interacting with reception
  Trusting the interpreter: sometimes patients want to bring a family member who can be problematic, especially if the family member is a child
  A few examples of interpreters ‘filtering’ what the patient says or being uncomfortable with sensitive topic
  Interpreter speaks a different language or dialect
- Patient experiences and expectations:
  High care needs of those with traumatic experiences and mental health conditions
  NHS more complicated than almost anywhere else, a ‘culture’ in its own right
  Sense of immediate urgency versus the appointment system/waiting for referrals
- Provider-related barriers:
  Perception of recent migrants as particularly demanding
  Awareness of eligibility for primary care services
- System-related barriers:
  Many recent migrants need more time and resources than other patients but there is no additional funding
  Rapidly changing and unclear regulations which leads to uncertainty about who is eligible for receiving free healthcare and whether ID should be requested
  Mobility of patients; hard to establish continuity of care
  Absence of medical records especially on medications taken

Facilitators for successful care

- Motivation and interest
  Need to really want to engage with migrant patients!
  Expertise in the practice, for example, in working with asylum seekers
  Some practitioners are motivated by their own experience of migration
  Spending time getting to know patients/making them feel welcome
- Taking time
  Understanding that taking time at the beginning will avoid problems later
  Taking time establishing medical history after registration
  Need to build trust around mental health/sensitive areas
  Successful consultations with an interpreter take a lot longer
  Establishing new migrant health check where more time is available
- Overcoming language barriers
  Members of staff with the same language or cultural background can be helpful but this can impact on workload and practitioner–patient dynamics
  Team-based approaches taken by practice to make best use of members of staff with language competencies
  Continuous relationship with trusted interpreter
  Use of bridging people—community health champions, link workers
  Strategies to check understanding and picking up misunderstandings, for example, on next consultation

## Results

### The core theme: a ‘fit’ between patients and practice

As outlined above, we found that most practices aimed to develop a fit between patients and practice. This could include a fit in language or cultural terms, in what has been conceptualised as ‘concordance’ mainly by US academics^21^ or a flexible approach and welcoming attitude expressed by the practice. While there were many different descriptions of what a good fit would look like, for some they encompassed good rapport and a shared ethos:

[O]ne of the nicest things in this practice is that the people who settle down with us, we understand them, they understand us, we appreciate them, they appreciate us, and I think that, kind of, harmonisation takes time, it's quite beautiful (GP, Practice 1).

However, another conceptualisation of a fit was as a match between what practice staff felt they were able to provide and recent migrants' expectations; which could lead to the perception that it was the responsibility of the patient to adapt to the practices' structures and resources.

### Theme 1: “A Pandora's box”—what migrants bring with them

This theme can be expressed in the following statement: *What migrant patients bring with them (or do not bring with them) may make it difficult to fit in with the way that primary care practices are organised*. Caring for recent migrants sometimes challenged practices because migrants lacked medical records detailing possibly serious care needs; this was especially true for migrants from low-income countries with a basic health service or from war-torn areas. The absence of records was important as practices increasingly relied on structured ways of delivering care with well-ordered records:

[W]hen you have a baby from another country, you don't have medical records, what do you do with that baby? … it's a Pandora's box—you open it but you don't know what is in it. Forget the language … forget the culture … they take an extra bit of time because the normal assumptions that we make in a clinical environment, in a consultation you can't make those assumptions (Admin staff, Practice 1).

The absence of records made it more difficult to continue or replace treatment patients had received before migrating to the UK. Several participants said that they struggled with identifying medications:

I saw one man not long ago, he is saying that he has diabetes and he is on medication and then he brought in his medication … some [patients] do bring [their medicines] with them then at least you can make sense what it is or you can just type the name and it will tell you what it is. Google … what would we do without Google? (laughs) (Nurse, Practice 1)

For some migrants, especially those from low-income countries, there was the possibility of wider care needs as yet undiagnosed, including unresolved problems that had not been properly treated in their country of origin:

[I]n some way one could argue that this [a broken arm that had not set properly] is slightly unfair for the NHS. You know this is problem that happened abroad; someone else didn't manage it very well and we now have to sort it out. … It is no fault of their own, it is just a situation [migrants] find themselves in (GP, Practice 9).

Previously undiagnosed mental health problems were said to demand a lot of time and resources. These could relate to trauma experienced by asylum seekers and refugees, or mental illness especially among women which was not recognised and might have been stigmatised in their own families and communities. Following their experience of complex health needs arising in a 10 min consultation, especially with asylum seekers and refugees, Practice 2 had instituted a system where new patients from countries suggesting that they might have unmet health needs were given appointments for a ‘new migrant check’. The nurse responsible for these checks discussed how finding out about these needs often took time and trust:

And if we register somebody who might be a vulnerable migrant … we ask them questions. It ranges from physical health problems, mental health problems, torture and it's very comprehensive. … Often they don't want to talk about some things in that first appointment because they want to get to know someone. It's usually a transitional thing and it takes sometimes one appointment and sometimes it's ten (Nurse, Practice 2).

Many participating GPs and nurses said that they tried to uncover physical and mental health needs to continue or start the appropriate treatment; some also felt that they would, in the long run, save time and resources that might be need to be mobilised later if problems were not addressed early. However, this was time and resource intensive and practices were not reimbursed for the extra work as patients with complex needs did not fit easily within the usual assumptions of structured records and short consultations.

### Theme 2: Reaching crisis point

The second statement (below) also expresses difficulties in connecting migrants to the work of the practice: *A perception that the practice is in crisis and demands are overwhelming can lead to practice staff insisting that migrants fit in with the practice*. Most of the participants held a balanced view on demands from migrant patients. However, a vocal minority of four participants based at practices 4, 6 and 8 strongly felt that their practice had reached crisis point where demands from migrants posed an unacceptable burden. They recounted experiences with patients ‘saying I want. … I want …’; ‘kicking off’; ‘waving their arms around’:

There's no recourse to people who abuse the system, who take no responsibility … and unfortunately, it falls upon the practice, your scores go down, the things that you are trying to achieve go down (GP, Practice 6).

The patients have been spoilt by our main GP … times are changing, this is what we have to say to them. If you want to see a doctor and we have to meet your demands, you will see any doctor. They really have now to mould to our service and this is what they are not understanding—they won't change (Admin staff, Practice 8).

Rather than trying to achieve a fit with patients, these participants decided that it was up to patients to fit in with the workings of the practice by becoming less demanding. Some also perceived that the NHS itself might be endangered by overly demanding migrant patients:

It needs to be done right at the beginning when people are registering to come into the country. … Go to a group session about how the NHS works. You know, why is it important to British people? You know, how it is struggling at the moment? (GP, Practice 6)

[The NHS] is such a beautiful, beautiful service we offer and it's just being abused with people's expectation … if we lose it, we lose more than we can ever imagine. You still have to keep the smile on your face and say we are here to help you, you know how can we help you? And sometimes you just want to throttle [patients] but you can't (Admin staff, practice 4).

Worry for the practice's welfare was increased by uncertainty about entitlements and liability or loss of money that might be incurred if they cared for a patient with unclear immigration status or without ID (proof of identity):

The migrant health worker [at a training event] said you shouldn't be asking for ID because that's a barrier. I kind of agree and I kind of disagree because we've had fraud, we've had problems with the police. And when they ask for an address and it's not a valid address we've got nothing to go on (GP, Practice 6).

We have to have proof of ID, we have to have proof of address. … If they are not residents of this country, we'd like to just see if they were to be registered in this country, … if there's anything on the passport that will say ‘not to be seen by some GP's’ [probably means ‘no recourse to public funds’] I have a look at that (Admin staff, practice 4).

These views need to be seen in the context of practice admin and reception staff being placed in a difficult position, having to control access to healthcare and making supply fit demand.^22^ However, it seems that demands from migrant patients were seen as more problematic by these participants as they threatened possible legal liability or additional costs which were viewed as endangering the viability of the practice. It is impossible to conclude whether these fears were related to personal experiences, difficulties encountered by particular practices or a wider systemic issue; we also do not know whether these views differed from those of practice colleagues that were not interviewed. However, all three practices were of small size and situated in areas of high diversity which may indicate that participants' feeling of crisis was linked to the lack of capacity in small surgeries to address complicated challenges such as understanding the entitlements of diverse patients.

### Theme 3: Speaking the same language

This theme is summarised as: *Speaking the same language can create a fit between patients and practice but may also become problematic*. Several participants discussed how speaking the same language created instant rapport: “The first thing is that they feel accepted and understood” (GP, Practice 9). Having this rapport could be instrumental, for example, for mental health:

My consultations were through someone who speaks Mirpuri and one of these things I soon learnt is that they would have a better rapport with the interpreter than me … in due course that person became so good at the consultation that we could identify a lot of people with depression and anxiety (GP, Practice 1).

Participants also mentioned examples for interpreted consultations being less than ideal (issues of trust and competence, patients preferring a family member as interpreter) which we touch on only briefly as these have been widely discussed in the literature.^7^ ^23^ ^24^

There was also an understanding in some practices that practice staff should be recruited from the wider community to provide language and cultural concordance: “[Receptionists] live among the community, they know the community and they speak the language and they understand the dynamic” (GP, Practice 9). However, for some, speaking the same language could become problematic. A second-generation migrant GP who could speak conversational Punjabi but was more comfortable conducting a medical consultation in English discussed his experience when his practice took over the patient list of an older colleague:

Their experience of primary care was, this is my doctor, when I turn up we have our consultation in Punjabi. … I start the consultation in English … they will start talking to me in Punjabi, now I feel that I have to kind of respond … all my schooling has been in the UK, my brain works in English. I can speak in Punjabi (GP, Practice 10).

Another possible problem was the blurring of boundaries and assumptions being made depending on a person's name or language competencies:

Patients will book with me, because they think [because of the GP's name] I will be able to speak their language and they're quite upset that I can't speak Urdu or Punjabi or Gujarati. … I don't know whether the doctors who speak the language are happy to see all these people … because sometimes [patients] feel the doctor speaks their language and the familiarity sometimes is in abundance, so they feel they can ask for more things (GP, Practice 3).

While the impact on workload is only alluded to in the above examples, staff at one practice reported major problems when a GP and receptionist spoke an in-demand language (not specified due to anonymisation) which attracted a lot of new patients:

She [receptionist] feels pressurised because if they feel or they get to know that somebody knows their language, that's it. A lot of the times she's in the back [of reception] but everyone will ask for her. … The doctor … is here all day, as soon as he finishes morning surgery, evening starts straightaway ‘cos it's so demanding at the moment (Admin staff, Practice 8).

The dynamics related to language were slightly different for more established or recent migrants. The first example illustrates how the retirement of the cohort of South Asian GPs who had joined the UK medical workforce brought a change from consultations with a personally known GP that spoke the same language.^25^ The other examples could refer to recent or established migrants; however, as recent migrants spoke a more diverse range of languages, it was less likely that they would find a language concordant GP (which could explain why increasing numbers of recent migrants registered with Practice 8).

Participants who felt that it was up to patients to create a better fit also stressed that they should do so by learning English. One practice manager felt strongly that patients should be able to speak English because using their first language in the practice allowed patients to become overfamiliar and leave her out of the loop:

No one wants to say, well actually it was your responsibility to learn a bit of English … if you can speak in your mother tongue and get a twenty minute appointment and not have to pay, what importance is there to learn that language and get ahead? (GP, Practice 6)

I feel that my staff are abused—[the receptionist] especially … it's my rule that you speak English because if she is being abused by a Punjabi speaking patient, I can't understand what the hell is going on (Admin staff, Practice 8).

These accounts show that although a shared language can be very positive, it was also perceived as problematic, blurring boundaries and perhaps enabling stronger demands to be made because of an assumed social bond. A more structured approach (eg, dedicated clinics in a particular language which could be booked) might have helped to mitigate these effects.

### Theme 4: Building bridges

This theme is summarised as: *A conscious effort to build bridges between practice and patients can be successful in creating a fit, thereby improving services*. Some practices described how they developed a clear, often team-based, strategy. Several PCPs were motivated by their own experiences or sense of justice in making sure migrants received appropriate care:

I was born in one continent and grew up in two others. So up to the age of 14 I was constantly changing cultures and always the outsider. … So I have natural patience and the heart for people who are the outsider (GP, Practice 9).

The really important thing which we haven't said is that you just have to want to do it and you have to recognise that it doesn't matter where people are born; they are people who deserve your full attention (GP, Practice 2).

One way of reaching out to migrant patients was the use of dedicated ‘bridging’ people who could be clinical staff, reception staff, link workers or translators. This worked best in larger practices where it was easier to make use of the skills of multilingual staff without overloading them:

We looked at the skills again and then we said, ‘you have to speak English, obviously, you have to speak a bit of Mirpuri or Urdu but you also need to speak Pashto’. … Fortunately for us we have people who are trilingual, so we have to, kind of, pass calls round in the practice to meet the needs (GP, Practice 1).

Participants from Practice 1 discussed language as a skill which could be learnt (starting with being able to say a greeting and a few welcoming phrases). Talking in the patient's own language to create rapport and a welcoming atmosphere was also seen as distinct from what was needed to discuss medical matters (where an interpreter might be appropriate). A skill-based approach was complemented by the use of dedicated link people to address social as well as clinical needs:

On Tuesday and Wednesday we have a mainly Romanian clinic and we have a fulltime interpreter there so when they come in, they feel welcome, someone speaks their language, we've also employed a part-time Romanian receptionist and in a baby clinic on Friday morning we also have a Romanian link worker (GP, Practice 1).

The above practice also purposefully embraced the concept of cultural competence as a necessary skill that could be acquired through training and practice. In this example, sensitivity to patients' core values and experiences was more important than adherence to ‘cultural norms’, for example, related to gender:

For example we had Pashto speaking women who are seeing an Afro-Caribbean mental health worker and asking for him by name … whereas if you normally went to this group and say who do you want to see? They will say ideally we would like to see a person who speaks our language who is female … yes? But because they have realised that this guy is competent their cultural norm is now on its head. (Admin staff, Practice 1)

## Discussion

This study's findings point to the vital role of making adjustments to usual ways of working and adopting a flexible approach to provide good care to migrants. However, another important finding was that some participants strongly believed that it was down to migrants to adjust and that trying to create a fit between practice and patients imposed an unacceptable burden. Through the force of circumstances rather than a disregard for conventions surrounding medical care in the UK, migrants may present challenges (no medical history, unresolved/undiagnosed health issues) that make it difficult for them to fit in with the workings of the practice (Theme 1). Staff in practices perceiving themselves to be at crisis point may put the onus on migrants to adapt to them (Theme 2). A shared language can contribute to achieving a fit, but can also be problematic if assumptions, for example, about social bonds created by language are not consciously addressed by practice staff (Theme 3); clearly articulated, team-based strategies to create bridges between practice and patients contributed to creating a better fit (Theme 4). A view of language and cultural competency as skills that can be learnt may be beneficial to avoid overloading bilingual members of staff; this may work better for larger practices where the team can cover a greater range of languages and other skills. Overall, participating practices could be characterised as one of three types: medium-sized to large-sized practices in very diverse areas who were proactive in engaging with recent migrants; small practices in very diverse areas where migrants were perceived as contributing to a sense of crisis and overload, and small to medium practices with low levels of diversity, where participants talked about a lack of experience in engaging with unfamiliar groups or entitlements or the necessity of preparing for the expected arrival of new migrants of yet unknown origin in their area.

We found that barriers to accessing healthcare were similar to those outlined by studies with a focus on marginalised and more vulnerable migrants, that is, communication difficulties, lack of awareness of migrants' health needs and impact on resources.^8^ ^26–28^ Mota *et al*^11^ also discuss facilitators (eg, motivation and feeling useful, experience of living and working overseas, enjoying the challenge) which resonate with our participants' accounts. While they discussed service-related and system-related barriers (access to interpreting services, awareness of eligibility for primary care services), a provider-related barrier also emerged in some participants' perception of recent migrants as an unacceptable burden on health services. The stigmatisation of refugees and asylum seekers accessing healthcare is well known;^7^ in our study, this stigma seems to have been extended to all recently arrived migrants. As refugees had reduced the number of visits to a GP to avoid being perceived as a burden,^7^ a similar pattern might occur in other migrant patients finding themselves stigmatised.

These findings should be seen in the context of wider, political developments. At the time of interviewing (2014–2015), migrant health was frequently in the news. A Home Office policy document had portrayed the use of NHS services as a major pull factor for migrants^29^ and argued for reducing their access to healthcare as a way of controlling immigration although there is no empirical support for that argument.^30^ An increasing focus on charging for services to migrants,^31^ combined with a general tendency towards excluding ‘outsiders’ from receiving health services in times of perceived duress,^32^ may have affected some participants' perception of demanding migrant patients as a major drain on the NHS. Confusion about entitlements and the need for ID are an ongoing issue in UK primary care. Although an NHS guideline states that the lack of ID should not be a barrier,^33^ vulnerable migrants are still being refused registration in primary care.^27^ ^34^ In parallel with a more restrictive stance in the UK and many European countries, there is also a Europe-wide drive to improve healthcare for migrants, with a growing evidence base outlining how this could work in practice; such as increasing cultural competence (an overall ethos of awareness and openness towards diversity rather than assuming that individual groups have a set of cultural beliefs or customs to which practitioners must respond).^35–38^ Their recommendations echo strategies already adopted by some practices.^35^ ^37^ It remains to be seen what the impact of the UK leaving the European Union will be on migrants' access to primary care services and the quality of encounters between migrants and PCPs.

Research by the King's Fund has shown that smaller practices are struggling with new clinical, administrative and regulatory demands frequently and find it difficult to develop formal links with other services.^39^ In our study, some of the participants based at small practices perceived providing healthcare to migrants as a drain on their resources reflecting findings from an earlier study focusing on refugees.^11^ Providing access to staff with shared languages might be beneficial to migrants^6^ but can place pressures on small practices if bilingual members of staff become overburdened. Clearly, a system is needed to ensure patients know when such members of staff are available. There were diverging opinions on who was responsible for creating a fit between patients and practice, with some practices very clearly seeing it as their role to reach out to patients while others thought that patients should adapt in order to fit in with the practice. The nature of what should be ‘fitted’ can be contested: a study from Belgium found health professionals saw overcoming language barriers as their responsibility but were less prepared to adapt to preferences they perceived as cultural.^40^

This study is, to the best of our knowledge, the first qualitative inquiry focusing on the perspectives of PCPs in the UK on their experience of providing healthcare for migrant patients which goes beyond those working with asylum seekers. However, there are limitations to the study: although appropriate for an exploratory qualitative design in primary care,^41^ the sample size is small; it became apparent that most of those who agreed to take part either were strongly motivated to provide good care to migrant patients or struggled with what they perceived as rising demands—a limitation which is common to most qualitative research.^42^

While recommendations based on our findings are tentative due to the exploratory nature of the study, some practices gave examples of good practice that may have potential for wider adoption. These included hiring culturally and linguistically diverse staff (with a clear strategy on how best to use their skills), and having designated days on for certain groups, for example, Romanian patients. Having members of staff with specific roles in caring for migrants (eg, doing new migrant checks) was also beneficial; this included link workers employed to build bridges to migrant patients. While participants argued time spent effectively identifying health needs would save resources in the long run, they also discussed the need for additional resources to provide services to the most vulnerable patients with great physical and mental health needs. A change in payment models allocating additional funds to practices providing care for these patients has long been proposed^11^ but is still not realised. The pressure that the current move to restricting migrants' access to healthcare puts on practices should also be acknowledged. Exhortations to remove barriers to access might have only limited success (as described above by one participant); struggling practices might benefit from non-judgmental support and easily accessed, up-to-date information on entitlements and charging regimes for migrant patients. Future research could explore the social and cultural dynamics that make a good fit, and attempt to compare the value of concordance-based approaches (centred on shared language or culture) versus competence-based approaches (focused on skills that can be learnt). Further research is also needed to unpick the reasons behind the strongly felt and worded negative views of migrant patients especially by practice receptionists and managers. A focus on the experience of administrative staff will be important as qualitative studies in primary care tend to centre on clinicians' views.

## Conclusions

The scale, speed and diversity of new migration bring a range of challenges. While participants agreed on a fit between patients and practice as desirable, some made changes to the services they provided to adapt to the needs of recently arrived migrants, while others thought that it was the responsibility of migrants to adapt to practice needs; a few viewed migrants as a burden to the system. Small practices without a clear strategy on how to work with an increasingly diverse patient population struggled most; those faring better had planned and developed a range of ways to engage with migrant patients. Practices might consider introducing ‘bridging’ roles to connect to particular communities; they could also aim to foster a general stance of openness to diversity.^43^
